# Rapid Response of *Daphnia magna* Motor Behavior to Mercury Chloride Toxicity Based on Target Tracking

**DOI:** 10.3390/toxics12090621

**Published:** 2024-08-23

**Authors:** Feihu Qin, Nanjing Zhao, Gaofang Yin, Tao Wang, Xinyue Jv, Shoulu Han, Lisha An

**Affiliations:** 1 University of Science and Technology of China, Hefei 230026, China; qinfeihu@mail.ustc.edu.cn (F.Q.); xinyuejv@mail.ustc.edu.cn (X.J.); lishaan@mail.ustc.edu.cn (L.A.); 2 Anhui Institute of Optics and Fine Mechanics, Hefei Institutes of Physical Science, Chinese Academy of Sciences, Hefei 230031, China; fhqin@aiofm.ac.cn (T.W.); hslo_jy@163.com (S.H.); 3 Key Laboratory of Optical Monitoring Technology for Environmental, Hefei 230031, China

**Keywords:** target tracking, *Daphnia magna*, toxic response, motor behavior, sensitive indexes

## Abstract

A rapid and timely response to the impacts of mercury chloride, which is indispensable to the chemical industry, on aquatic organisms is of great significance. Here, we investigated whether the YOLOX (improvements to the YOLO series, forming a new high-performance detector) observation system can be used for the rapid detection of the response of *Daphnia magna* targets to mercury chloride stress. Thus, we used this system for the real-time tracking and observation of the multidimensional motional behavior of *D. magna*. The results obtained showed that the average velocity ($\bar{v}$), average acceleration ($\bar{a}$), and cumulative travel (L) values of *D. magna* exposed to mercury chloride stress changed significantly under different exposure times and concentrations. Further, we observed that $\bar{v}$, $\bar{a}$ and L values of *D. magna* could be used as indexes of toxicity response. Analysis also showed evident *D. magna* inhibition at exposure concentrations of 0.08 and 0.02 mg/L after exposure for 10 and 25 min, respectively. However, under 0.06 and 0.04 mg/L toxic stress, $\bar{v}$ and L showed faster toxic response than $\bar{a}$, and overall, $\bar{v}$ was identified as the most sensitive index for the rapid detection of *D. magna* response to toxicity stress. Therefore, we provide a strategy for tracking the motile behavior of *D. magna* in response to toxic stress and lay the foundations for the comprehensive screening of toxicity in water based on motile behavior.

## 1. Introduction

Ensuring the ecological and environmental safety of water is of great importance. However, owing to rapid advances in industrial and agricultural development, trace metals have been released into the air, water, and soil via various means [1,2]. These pollutants are characterized by poor degradability, low mobility, and easy accumulation, as they readily accumulate in the environment, posing various ecological risks [3]. Additionally, it has been demonstrated that trace metals can be absorbed by plants, accumulate in animals, and enter the human body through the food chain [4]; this causes carcinogenic and teratogenic diseases and damages the nervous, digestive, reproductive, and immune systems [5]. Specifically, mercury, a heavy metal with high toxicity, is a main pollutant that can potentially compromise the health of aquatic ecosystems if its threshold concentration is exceeded [6], and it exerts several effects on aquatic biota, ranging from developmental and reproductive toxicity to neurotoxicity [7]. It has also been shown that mercury ingested by human is oxidized in the gastrointestinal tract to mercury salts (e.g., such as mercury chloride), which show increased circulatory absorption in the body and can further exert toxic effects on the central nervous system, gastrointestinal tract, and kidneys [8]. Mercury chloride, one of the extremely toxic mercury salts, is an important disinfectant and raw chemical material [9,10], and its production and utilization inevitably result in leakage into the water environment. It has also been shown that even at low concentrations, mercury can adversely affect the growth, development, and reproduction of marine organisms [11]. Therefore, there is an urgent need to develop a fast and effective strategy for evaluating the biological toxicity of mercury chloride.

*Daphnia magna*, a primary consumer in the food chain, is often used as a model organism in toxicological research owing to its large body size, reproductive capacity, and ability to reproduce asexually via parthenogenesis [12,13]. Its sensitivity to various environmental stressors in aquatic ecosystems [14,15,16,17] also makes it ideal for studying aquatic biotoxicity. Thus, it has been used in several studies on metal toxicity in aquatic environments [18,19].However, traditional acute toxicity endpoint assessments, which are typically based on mortality or immobilization experiments, are often less sensitive and are also time-consuming [20]. Thus, they are unsuitable for assessing the sublethal impacts of low-concentration contaminants in water bodies. Therefore, more sensitive biomarkers are required for the comprehensive analysis of the toxic effects of pollutants, especially those of low-concentration pollutants. Studying the effects of the short-term exposure of aquatic organisms to these pollutants is also of great significance.

A previous study by Melvin and Wilson revealed that to assess the toxicological effects of environmental contaminants, behavioral studies are fast, sensitive, and powerful tools for toxicological studies [21]. Additionally, the behavioral responses of the embryos of zebrafish, owing to exposure to chemical stress, indicate that biobehavioral stress often precedes death and that the concentration of a behavioral stressor for the same exposure time is significantly lower than the lethal concentration [22]. Further, given that the biological movement behavior shows rapid response to toxic stress, it has potential as an early warning signal for water pollution; hence, it can be used as a tool to evaluate the toxicological effects of environmental pollutants. In recent years, sensitive biomarkers, such as swimming behavior, have attracted considerable attention from scientists [23,24,25]. To clearly and intuitively evaluate the degree of the influence of toxic stress on *D. magna*, biomarkers such as swimming behavior are generally used for quantitative analysis. Studies have also shown that pesticides and heavy metals such as copper can inhibit the swimming speed of *D. magna* [25,26], which is generally expressed as the average velocity index. The use of acceleration indicators related to swimming speed to study the effects of toxic substances on motility behavior has also been reported [23]. However, swimming behavior is performed with the use of some biomarkers related to this parameter, and differences in toxicity response between these different indicators remain unresolved. Therefore, it is necessary to further study the toxicity response of the multidimensional motional behavior of *D. magna*.

Presently, environmental risk assessment methods for water quality are primarily based on the evaluation of toxicity levels based on acute toxicity tests using aquatic organisms. Even though the response of the motility of *D. magna* to stress is critical in assessing the environmental risks of new pollutants and neuroactive drugs [24,27], it has not been used in routine biotoxicity tests with reference to stressors, and studies on rapid behavioral responses to short-term exposure to toxic stress are limited. Mercury chloride is chemically stable after dissolution in water and is often used as a reference toxicant in acute toxicity testing for luminescent bacteria (ISO-11348-3; GB/T15441-1995) [28,29]. Generally, the endpoint for determining acute toxicity in such tests is typically based on the lethal effects of this chemical on organisms. The commonly used parameter is LC_50_, which is the concentration at which a substance causes death in 50% of the test organisms within 24 or 48 h [30]. However, xenobiotic (mercury) has been shown to induce oxidative stress in cells, leading to disease, aging, and cell death [31]. Furthermore, mercury can have a negative impact on the behavior and health status of tropical freshwater fish [32]. These results indicate that heavy metals have adverse effects on the mobilization of *D. magna* by generating reactive oxygen species (ROS) [31]. The YOLOX is an improved version based on YOLO (You Only Look Once) for real-time object detection. Therefore, in this study, we constructed a *D. magna* target tracking observation system based on YOLOX and used it for the rapid observation of the response of *D. magna* motion behavior of the toxic stressor mercury chloride. Further, we observed multidimensional motion parameters, namely cumulative travel (L), average velocity ($\bar{v}$), maximum velocity ($v_{max}$), average acceleration ($\bar{a}$), and maximum acceleration ($a_{max}$), under different exposure times within a certain concentration range, and thereafter, we analyzed behavioral response patterns before the death of the organisms. Therefore, the aim of this study was to explore the potential of behavioral parameters as early warning indicators of environmental risks. We also aimed to accumulate technical knowledge regarding the rapid detection of integrated toxicity in water bodies.

## 2. Materials and Methods

### 2.1. Materials and Study Species

Female *D. magna* (aged ≤24 h) were purchased from the Guangdong Laboratory Animal Monitoring Institute (Guangzhou, China) and continuously cultured in a medium prepared according to the standard of the American Society of Testing and Materials (ASTM, 1986) for >2 months. *D. magna* culturing was performed in a thermostatic light box with the temperature maintained at 22 ± 1 °C and with the light–dark cycle set at 16 h light/8 h dark. Further, the test organisms were regularly fed with algae, *Chlorella* sp., at a density of approximately 10^5^ cells/mL. Then, to carry out the stress interference experiments, adult *D. magna* were separated from their younger counterparts using a nylon sieve (diameter 1 × 1 mm) and placed in filtered tap water (pH, 7.0–8.5; dissolved oxygen, >4 mg/L; hardness, 250 ± 22 mg/L) that had been aerated for >48 h for breeding. After a breeding cycle of not more than 24 h, the nylon sieve was again used for the second screening to remove the adult *D. magna* and obtain juvenile *D. magna* (<24 h), which were then used for the behavioral assays. The reference toxicant was mercury chloride (Sigma-Aldrich, St. Louis, MO, USA), and its concentration range was selected according to the standard of acute toxicity equivalent to luminescent bacteria (ISO-11348-3; GB/T15441-1995).

### 2.2. Experimental Design

**Figure 1:**
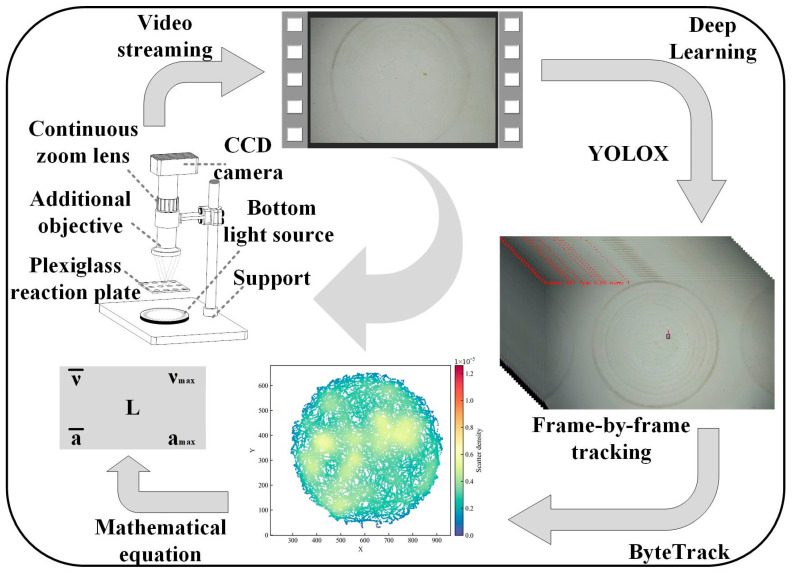
A schematic representation of the experimental observation system.

A schematic representation of the observation system used in this study is shown in Figure 1. In brief, the observation system consisted of a charge-coupled device (CCD) camera, a continuous zoom lens, a U-shaped plexiglass reaction plate, an LED bottom light source, and an additional objective lens module. The behavioral movement of *D. magna* was recorded at 30 fps using the CCD camera, and the videos obtained were stored on a computer for subsequent analysis. 

When using video target recognition and tracking algorithms, the movement and changes in the pose of plankton can cause target tracking failures, resulting in missing or unwanted frames [33], and an extremely short calculation period may lead to inconsistent tracking of frame numbers, resulting in inaccurate cumulative travel. Therefore, it is more reliable to use a period of 5 min to determine the indicators of the motion behavior of *D. magna*. In this study, different concentrations of mercury chloride (0 (control), 0.02, 0.04, 0.06, and 0.08 mg/L) were employed. Before the toxicity stress experiment, 60 juveniles from the same batch were screened and transferred onto porous (25 mm diameter) plexiglass reaction plates for behavior observation. The juveniles were first allowed for environmental adaptation for 5 min before the observation. Thereafter, similar experiments were performed using the different concentrations of mercury chloride for 30 min. Twelve parallel experiments were performed for each concentration. 

### 2.3. Data Processing

Presently, You Only Look Once (YOLO) is the most widely used object detection algorithm series. Its main feature is its fast detection speed, and over the years, its detection accuracy, known as mAP, has also been improved to obtain high-performance YOLOX [34]. Further, another multi-target tracking algorithm series, ByteTrack, combines target detection and target tracking by associating each detection box rather than by simply reaching a conclusion solely based on a high-score detection box. This approach circumvents issues related to target occlusion (e.g., missed detection and fragmented trajectory) and greatly improves the accuracy and efficiency of the algorithm series [35]. Additionally, ByteTrack has a lightweight design that contributes to its excellent performance. Therefore, in this study, we combined YOLOX and ByteTrack as the final scheme for the *D. magna* target. Specifically, our video processing primarily relied on the YOLOX object detection algorithm and the ByteTrack object tracking algorithm to continuously track targets of *D. magna* motion video data and output the coordinate information regarding each target frame.

Treating the moving *D. magna* as a moving point target, the recorded video could be processed using the target tracking algorithm in the observation system to obtain the coordinate data (*x_i_*, *y_i_*) of the target in each frame. Further, to characterize the motion behavior of *D. magna*, the index parameters were obtained based on the continuously tracked target coordinates. *i* represents the frame number of the recorded video, and (*x_i_*, *y_i_*) represents the plane coordinates of the target recognized in the *i*-th frame. L represents the cumulative travel of the target obtained by summing the distances covered by *D. magna* in the front and back frames of a video. $\bar{v}$ represents the average velocity of *D. magna* calculated as the ratio of the cumulative travel to the duration of the video segment (5 min), and $v_{max}$ is a measure of the speed difference between the front and back frames and represents the maximum instantaneous speed in the cycle. Further, $\bar{a}$ and $a_{max}$ represent the average value of the cumulative change in the speed of the target in the preceding and following frames and the maximum change in speed, respectively.

For the purpose of this study, the five motor behavior indexes of *D. magna* were calculated by Python. All data were processed by IBM SPSS Statics 19.0 using a statistical criterion of *p* < 0.05, and the average values were presented. Normal distribution and homogeneity of data were evaluated by the Kolmogorov–Smirnov test and Levene’s test, respectively. In this study, one-way analysis of variance (ANOVA), followed by Tukey’s post hoc test, was performed to compare the effects of the exposure experiment on the motor behavior of *D. magna* with the controls. 

## 3. Results and Discussion

### 3.1. Observation Results under Controlled Conditions

**Figure 2:**
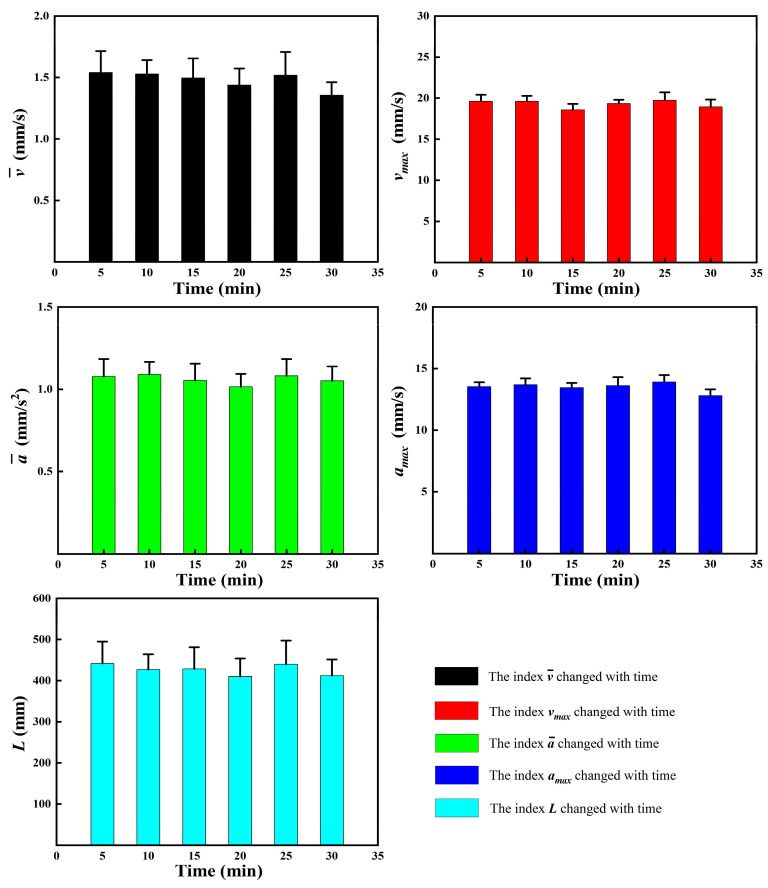
Motor behavior indexes (L, $\bar{v}$, $v_{max}$, $\bar{a}$, and $a_{max}$) of *D. magna* showing no significant changes within a 30 min observation period. The results are expressed as mean ± SE.

Observation experiments were conducted without toxic stress to determine the effects of the observation system on the motility response of *D. magna*. The observation period included six 5 min segments, making a total of 30 min. During this period, the mobility behavior of *D. magna* was monitored and analyzed under different concentrations of mercury chloride. The five indicators used to characterize the motion behavior of *D. magna* within the 30 min are presented in Figure 2. We noted that the differences between five behavioral motility indicators (L, $\bar{v}$, $v_{max}$, $\bar{a}$, and $a_{max}$) of *D. magna* during the observation period were not significant, and the indicators showed no significant downward or upward trends. This is in good agreement with the results of previous studies [36]. So, it was considered suitable for studying the motility behavior of *D. magna* following exposure to toxic stress. 

### 3.2. Observation Results under Toxic Stress Conditions

**Figure 3:**
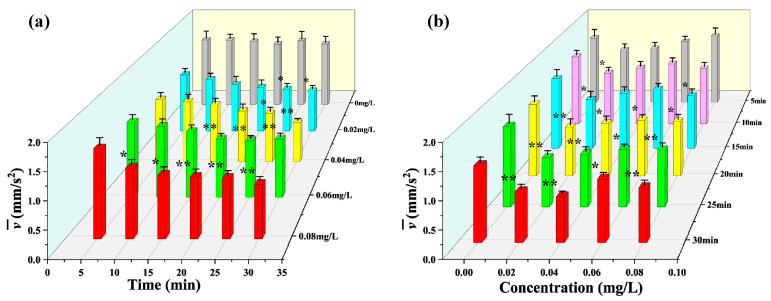
Average velocity $\bar{v}$ of *D. magna* exposed to different concentrations of mercury chloride and significant difference analysis compared to the control during the experimental period. (**a**) the average velocity $\bar{v}$ of *D. magna* with time under different mercury chloride concentrations; (**b**) the variation in the average velocity $\bar{v}$ of *D. magna* with mercury chloride concentration. The results are expressed as mean ± SE. * represents significant differences (*p* < 0.05), and ** represents highly significant differences (*p* < 0.01).

**Figure 4:**
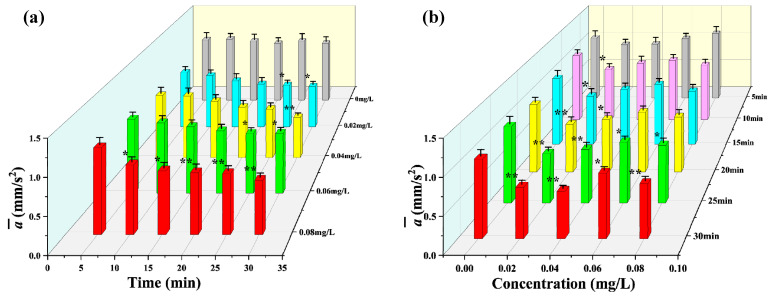
Average acceleration $\bar{a}$ of *D. magna* exposed to different concentrations of mercury chloride and significant difference analysis compared to the control during the experimental period. (**a**) average acceleration $\bar{a}$ of *D. magna* with time under different mercury chloride concentrations; (**b**) the variation in the average acceleration $\bar{a}$ of *D. magna* with mercury chloride concentration. The results are presented as mean ± SE. * represents significant differences (*p* < 0.05), and ** represents highly significant differences (*p* < 0.01).

**Figure 5:**
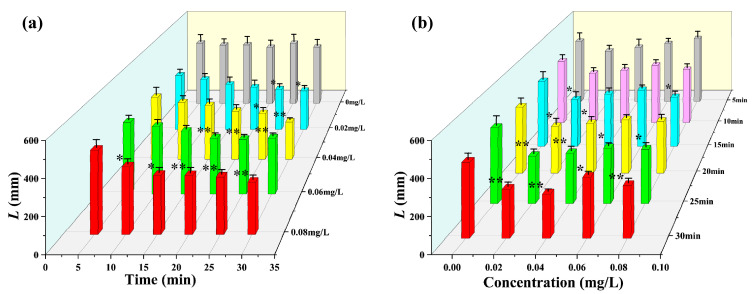
Cumulative travel L of *D. magna* exposed to different concentrations of mercury chloride and significant difference analysis compared to the control during the experimental period. (**a**) cumulative travel L of *D. magna* with time under different mercury chloride concentrations; (**b**) the variation in the cumulative travel L of *D. magna* with mercury chloride concentration. The results are expressed as mean ± SE. * indicates significant differences (*p* < 0.05), and ** indicates highly significant differences (*p* < 0.01).

**Figure 6:**
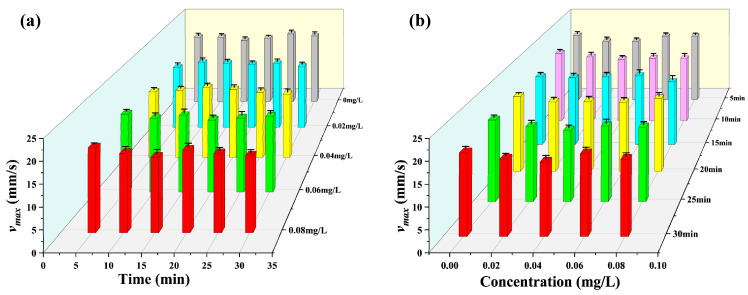
Maximum velocity $v_{max}$ of *D. magna* exposed to different concentrations of mercury chloride and significant differences analysis compared to the control during the experimental period. (**a**) Maximum velocity $v_{max}$ of *D. magna* with time under different mercury chloride concentrations; (**b**) the variation in the Maximum velocity $v_{max}$ of *D. magna* with mercury chloride concentration. The results are expressed as mean ± SE.

**Figure 7:**
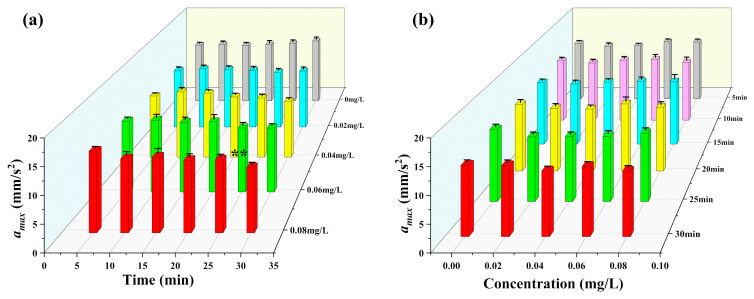
Maximum acceleration $a_{max}$ of *D. magna* exposed to different concentrations of mercury chloride and significant difference analysis compared to the control during the experimental period. (**a**) Maximum acceleration $a_{max}$ of *D. magna* with time under different mercury chloride concentrations; (**b**) the variation in the Maximum acceleration $a_{max}$ of *D. magna* with mercury chloride concentration. The results are expressed as mean ± SE. ** represents highly significant differences (*p* < 0.01).

According to the experimental design, the test organisms were exposed to different concentrations of mercury chloride, and then the target was continuously tracked for 30 min. Based on the statistical period of 5 min, the changes in the multidimensional motile indexes of *D. magna* were analyzed during exposure. The results obtained are shown in Figure 3, Figure 4, Figure 5, Figure 6 and Figure 7.

Figure 3a shows the average velocity $\bar{v}$ of *D. magna* with time under different mercury chloride concentrations. From this figure, it is evident that at the four different mercury chloride concentrations, the $\bar{v}$ values of *D. magna* varied significantly with increasing stress exposure duration; the highest concentration was 0.08 mg/L, which showed an inhibition response after 10 min of exposure. At a mercury chloride concentration of 0.06 mg/L, an obvious inhibition response was observed after 20 min exposure. However, under 0.04 mg/L and 0.02 mg/L of mercury chloride stress, no significant changes in $\bar{v}$ were observed until after 25 min of exposure.

Figure 3b shows the variation in the average velocity $\bar{v}$ of *D. magna* with mercury chloride concentration: it is evident that the $\bar{v}$ of *D. magna* only changed slightly with increasing the mercury chloride concentration. Regardless, the $\bar{v}$ showed potential as an effective index for the analysis of the rapid response of *D. magna* to toxic stress. After 5 min of exposure, there was no significant difference between the $\bar{v}$ values of *D. magna* and the blank group under different stress concentrations. However, when the stress concentration was 0.02 mg/L, we observed a statistically significant difference in response after 10 min of exposure, while other concentrations showed no statistical difference from the blank group. When the stress concentration was 0.02 mg/L and 0.08 mg/L, we observed a statistically significant difference after 15 min of exposure. Until 20 min after exposure, significant differences were observed in all stress concentrations compared with the blank group.

Studies have shown that the activity of acetylcholinesterase (AChE) in zebrafish brain is changed by heavy metal exposure, such as exposure to mercury chloride, which significantly reduces the activity of AChE and the antioxidant capacity of cells [37]. AChE is the key enzyme used to hydrolyze the neurotransmitter acetylcholine in the cholinergic synapses of vertebrates and invertebrates, and the inhibition of AChE can lead to decreased swimming ability, which is one of the main factors that changes the swimming behavior of *D. magna* [38]. Therefore, the *D. magna* exposed to mercury chloride also inhibited AChE, which caused the average velocity $\bar{v}$ of model organisms to decrease. 

As either the toxicant concentration or exposure time increased, a series of regulatory behavioral stress responses were activated and performed by the organisms. The behavioral strength of *D. magna* exposed to organophosphorus insecticides (OPs) demonstrated the presence of the stepwise stress model (SSM) [38]. According to our exposure experiment data, under the same exposure time, the average velocity $\bar{v}$ of *D. magna* decreased first, then increased, and then decreased with the increase in mercury chloride concentration. We found that the behavioral change trends of *D. magna* were consistent with SSM, which proved the SSM for mercury chloride in *D. magna*.

Comparative analysis based on the results presented in Figure 3a,b revealed that the changes in the average velocity $\bar{v}$ of *D. magna* were jointly affected by two factors, exposure time and stress concentration, and when the exposure time reached 30 min, the $\bar{v}$ values obtained were significantly different relative to those obtained in the blank experiment regardless of the stress concentration. Therefore, the main factor influencing the toxic response of *D. magna* under different toxic stress conditions in terms of $\bar{v}$ was the exposure time.

Figure 4a shows the significance analysis of the average acceleration $\bar{a}$ values of *D. magna* under different mercury chloride concentrations with time. From this figure, it is evident that the $\bar{a}$ values of *D. magna* changed significantly as the exposure time increased, and under the highest stress concentration investigated (0.08 mg/L), a significant inhibitory response appeared after 10 min of exposure. After exposure to 0.06 mg/L and 0.02 mg/L stress concentrations for 25 min, the average acceleration $\bar{a}$ showed a significant difference. While exposed to a mercury chloride concentration of 0.04 mg/L, a significant inhibitory response was observed after 30 min. Additionally, when the exposure time reached 30 min, the $\bar{a}$ values obtained under the different stress concentrations were significantly different from those obtained in the blank treatment.

Figure 4b shows the variation in the average acceleration $\bar{a}$ of *D. magna* with changes in concentration under the same exposure time. After 5 min of exposure, the $\bar{a}$ was not statistically different from the blank treatment at any of the four stress concentrations. However, when the exposure time reached 10 and 15 min, there was a significant difference between average acceleration $\bar{a}$ and blank treatment at the 0.02 mg/L concentration of stress. Furthermore, when the exposure time reached more than 25 min, the $\bar{a}$ of *D. magna* under all the exposure concentrations showed a significant inhibitory effect relative to the value obtained in the blank treatment. Comparative analysis of the data presented in Figure 4a,b further revealed that the main factor influencing the average acceleration $\bar{a}$ of *D. magna* under the different stress concentrations investigated was the exposure time. In addition, under stress conditions, the toxic response of average acceleration $\bar{a}$ was basically the same as that of average velocity $\bar{v}$, and the change trend of the two motion indicators was similar.

As shown in Figure 5a, the cumulative travel L of *D. magna* under the different stress concentrations changed significantly as the exposure time increased. From this figure, it is evident that at the four different mercury chloride concentrations, the L of *D. magna* varied significantly with increasing stress exposure duration; the highest concentration was 0.08 mg/L, which showed an inhibition response after 10 min of exposure. At a mercury chloride concentration of 0.06 mg/L, an obvious inhibition response was observed after 20 min of exposure. However, under 0.04 mg/L and 0.02 mg/L mercury chloride stress, no significant changes in L were observed until after 25 min of exposure. Further, from Figure 5b, which shows the significance analysis of the changes in the L of *D. magna* with concentration under the same exposure time, it is evident that the L of *D. magna* changed slightly with increasing stress concentration. Within the first 10 min of exposure, we did not observe any significant differences in the L of *D. magna* under the different concentrations relative to the value obtained for the blank treatment. However, after 15 min of exposure, significant differences in L were observed even under the lowest stress concentration (0.02 mg/L). Similarly, those were basically identical for the average velocity $\bar{v}$, average acceleration $\bar{a}$, and cumulative travel L toxicity response, and the change trend was also similar to that under stress conditions.

Figure 6a,b show the analysis of the maximum velocity $v_{max}$ of *D. magna* movement under different mercury chloride stress concentrations. From this figure, it is evident that at the four different mercury chloride concentrations, the maximum velocity $v_{max}$ of *D. magna* showed no significant differences during the stress period. Figure 7a,b showed that except for the highest stress concentration (0.08 mg/L), under which the maximum acceleration $a_{max}$ value of *D. magna* changed significantly after 30 min of exposure, the other stress conditions showed no significant differences. Although the $a_{max}$ is not a sensitive endpoint (compare to others), an increase in the concentration or exposure time may provoke a significant response. This situation may be due to the damage of the cell membranes of muscle cells after exposure to heavy metals [31], which leads to the destruction of muscle tissue and affects muscle strength and efficiency. Overall, this research suggests that the $v_{max}$ and $a_{max}$ biomarkers may not be reliable for toxic analysis of mercury chloride in the context of our study.

Relevant research has shown that mercury can affect AChE activity in vivo, lead to biological neurotoxicity [37], and impair biological motor functions [39]. Exposure to mercury chloride may cause anxiety-related responses, such as short- and long-term memory impairment, as well as motor deficits. Mercury can also accumulate in the hippocampus and cerebral cortex of organisms and promote functional impairment [40]. Additionally, studies have shown that changes in motility behavior of *D. magna* may also be caused by metabolic disorders [20], ion channel dysfunction [41], or muscle injury caused [42] by external stress. Mercury chloride can be toxic to macromolecules of organisms, inhibit enzyme activity, and damage the nervous system through a variety of mechanisms. Therefore, the experimental results showed that the motility behavior of Daphnia magna exposed to mercury chloride was inhibited. Previous studies have shown that inhibition of acetylcholinesterase is one of the main factors that changes the swimming behavior of *D. magna* after exposure to dichlorvos [38], and there is a relatively close relationship between the inhibition of AChE activity and altered behavioral parameters (feeding rate and motor behavior) [43]. Therefore, the motility behavior of *D. magna* is affected by exposure to mercury chloride, which is largely due to the inhibition of AChE.

Based on our experimental results, the motor behavior of *D. magna* under toxic stress changed, suggesting that its motor neurons were impaired. Therefore, based on the above results of our exposure experiments, $\bar{v}$, $\bar{a}$, and L can be used as sensitive indicators of toxicity response. However, the differences in response speed between these three motility indexes of *D. magna* can be further used to determine the optimal sensitivity index that allows for the rapid detection of responses to toxic species.

### 3.3. Comparison of Different Indicators with Respect to Toxicity Response

The results of the mercury chloride exposure tests showed that the $\bar{v}$, $\bar{a}$, and L of *D. magna* changed significantly under the combined influence of exposure time and exposure concentration. This observation indicated that the three motion indicators have potential for use as effective indicators for analyzing the rapid response of *D. magna* to toxic stress. However, differences in response characteristics between these three indicators still require further analysis.

**Figure 8:**
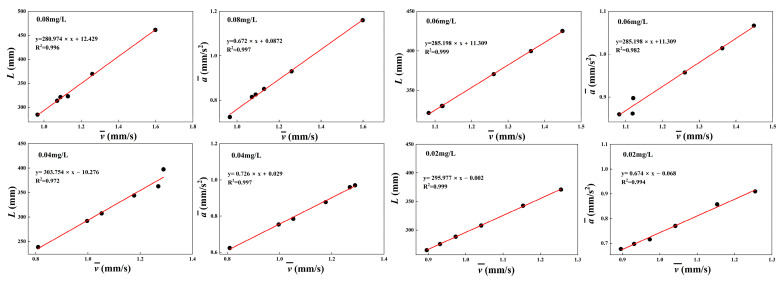
Correlation analysis of average velocity $\bar{v}$ with cumulative travel L and average acceleration $\bar{a}$ at different exposure concentrations. The results are expressed as means. A correlation study was performed.

Figure 8 shows the correlation analysis between the average velocity $\bar{v}$, cumulative travel L, and average acceleration $\bar{a}$, respectively, and the results show that there is a good linear relationship between the three aspects. Based on the linear relationship between the parameters, the conversion between the parameters can be realized. This phenomenon corresponds to the mathematical formula between velocity, acceleration, and accumulated travel. 

**Figure 9:**
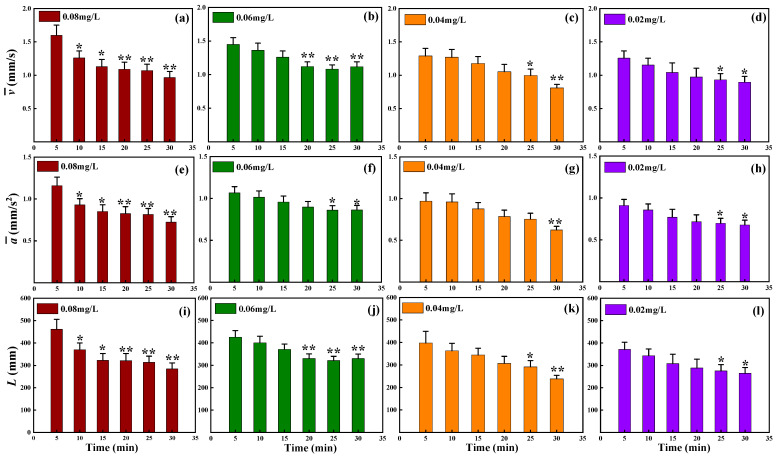
Comparison of three indicators ($\bar{v}$, $\bar{a}$, and L) of stress response and significant differences analysis compared to control. Average velocity $\bar{v}$ of *D. magna* with time at the mercury chloride concentration of (**a**) 0.08, (**b**) 0.06 (**c**) 0.04 and (**d**) 0.02 mg/L; average acceleration $\bar{a}$ of *D. magna* with time at the mercury chloride concentration of (**e**) 0.08, (**f**) 0.06 (**g**) 0.04 and (**h**) 0.02 mg/L; cumulative travel L of *D. magna* with time at the mercury chloride concentration of (**i**) 0.08, (**j**) 0.06 (**k**) 0.04 and (**l**) 0.02 mg/L. The results are expressed as mean ± SE. * represents significant differences (*p* < 0.05), while ** represents highly significant differences (*p* < 0.01). Further, the colored bars represent different exposure concentrations.

Figure 9a,e,i and Figure 9d,h,l show the same level of stress response for the three sensitivity indexes at exposure concentrations of 0.08 and 0.02 mg/L. However, it can be seen in Figure 9b,f,j that when the exposure concentration was 0.06 mg/L, the average velocity $\bar{v}$ and cumulative travel L showed extremely significant differences after 20 min of exposure, while the average acceleration $\bar{a}$ remained unchanged. Additionally, Figure 9c,g,k shows that when the exposure concentration was 0.04 mg/L, $\bar{v}$ and L showed significant differences after 25 min of exposure, while $\bar{a}$ still showed no significant change.

Even though the three indicators of *D. magna* response to stress showed significant changes within 30 min of exposure, further comparison revealed that the average velocity $\bar{v}$ and cumulative travel L could respond more quickly under the same stress conditions. Thus, we identified them as more sensitive motion indicators. In order to efficiently and comprehensively assess toxicity responses for accurate water quality evaluation based on the movement behavior of *D. magna*, the use of the more sensitive and effective indicators $\bar{v}$ and L of *D. magna* response to toxic stress is highly recommended. However, for the L indicator, it is susceptible to the impact of the target tracking process, especially during short-term exposure. Further, occasional loss of target tracking may lead to the final statistical cumulative distance results being inaccurate [33]. Conversely, the use of $\bar{v}$, which is based on the average of multiple measurements over a period, can significantly improve the bias caused by the accumulation of local travel. At the same time, average speed is one of the most reliable and widely applied parameters for biomarkers of *D. magna* sensitivity toxicity [20]. Taken together, the $\bar{v}$ of *D. magna* can serve as the most ideal indicator of response to the stress of toxicity.

## 4. Conclusions

In this study, we developed a real-time tracking method for *D. magna* based on the YOLOX object recognition algorithm, enabling the continuous monitoring of multiple dimensions of *D. magna* locomotion behavior owing to toxic stress. We effectively validated the rapid response of *D. magna* locomotion behavior to mercury chloride toxicity in short-term exposure experiments. Relative to the limitations of traditional acute toxicity tests (e.g., they require 24 or 48 h and are cumbersome), in this study, we demonstrated the monitoring of *D. magna* locomotion behavior as a strategy for rapidly realizing toxicity response tests. Statistical analysis based on two factors, exposure time and exposure concentration, indicated that short-term exposure to toxic stress could lead to significant changes in the $\bar{v}$, $\bar{a}$, and L values of *D. magna*. Further, by comparing the toxic responses of *D. magna* based on these three indicators, we identified $\bar{v}$ as an optimal indicator for the rapid detection of the response of *D. magna* to toxic stress. The rapid response of *D. magna* locomotion behavior to mercury chloride toxicity observed in this study via target tracking could provide a more comprehensive understanding of the toxic effects of aquatic pollutants. Additionally, this strategy lays the foundations for the rapid detection and screening of comprehensive toxicity in water based on motor behavior. Therefore, the real-time tracking and monitoring of model organisms not only serves as a high-throughput screening tool but may also be applied to online water environment risk early warning, significantly improving the efficiency and accuracy of environmental monitoring.

## Data Availability

The data that support the findings of this study are available from the corresponding author upon reasonable request.

## References

[1] Guan Q., Wang L., Pan B., Guan W., Sun X., Cai A. (2016). Distribution features and controls of heavy metals in surface sediments from the riverbed of the Ningxia-Inner Mongolian reaches, Yellow River, China. Chemosphere.

[2] Järup L. (2003). Hazards of heavy metal contamination. Br. Med. Bull..

[3] Chen J., Yuan L., Zhang Y., Xue J., Yang B., Wu H. (2023). Risk assessment of trace metal(loid) pollution in surface water of industrial areas along the Huangpu River and Yangtze River Estuary in Shanghai, China. Reg. Stud. Mar. Sci..

[4] Gall J.E., Boyd R.S., Rajakaruna N. (2015). Transfer of heavy metals through terrestrial food webs: A review. Environ. Monit. Assess..

[5] Briffa J., Sinagra E., Blundell R. (2020). Heavy metal pollution in the environment and their toxicological effects on humans. Heliyon.

[6] Cui L., Gao X., Wang Y., Zhang H., Lv X., Lei K. (2023). Salinity-dependent aquatic life criteria of inorganic mercury in coastal water and its ecological risk assessment. Environ. Res..

[7] Scheuhammer A.M., Meyer M.W., Sandheinrich M.B., Murray M.W. (2007). Effects of environmental methylmercury on the health of wild birds, mammals, and fish. AMBIO.

[8] Sandborgh-englund G., Einarsson C., Sandström M., Ekstrand J. (2004). Gastrointestinal Absorption of Metallic Mercury. Arch. Environ. Health Int. J..

[9] Clarkson T.W., Magos L., Myers G.J. (2003). The Toxicology of Mercury—Current Exposures and Clinical Manifestations. N. Engl. J. Med..

[10] Liu J., Shi J.-Z., Yu L.-M., Goyer R.A., Waalkes M.P. (2008). Mercury in Traditional Medicines: Is Cinnabar Toxicologically Similar to Common Mercurials?. Exp. Biol. Med..

[11] Wang M., Tong Y., Chen C., Liu X., Lu Y., Zhang W., He W., Wang X., Zhao S., Lin Y. (2018). Ecological risk assessment to marine organisms induced by heavy metals in China’s coastal waters. Mar. Pollut. Bull..

[12] Ebert D. (2022). Daphnia as a versatile model system in ecology and evolution. EvoDevo.

[13] Koivisto S. (1995). Is *Daphnia magna* an ecologically representative zooplankton species in toxicity tests?. Environ. Pollut..

[14] Biesinger K.E., Christensen G.M. (1972). Effects of Various Metals on Survival, Growth, Reproduction, and Metabolism of *Daphnia magna*. J. Fish. Res. Board Can..

[15] He Q., Wang X., Sun P., Wang Z., Wang L. (2015). Acute and chronic toxicity of tetrabromobisphenol A to three aquatic species under different pH conditions. Aquat. Toxicol..

[16] Kim Y., Choi K., Jung J., Park S., Kim P.-G., Park J. (2007). Aquatic toxicity of acetaminophen, carbamazepine, cimetidine, diltiazem and six major sulfonamides, and their potential ecological risks in Korea. Environ. Int..

[17] Wang X., Qu R., Liu J., Wei Z., Wang L., Yang S., Huang Q., Wang Z. (2016). Effect of different carbon nanotubes on cadmium toxicity to *Daphnia magna*: The role of catalyst impurities and adsorption capacity. Environ. Pollut..

[18] Altshuler I., Demiri B., Xu S., Constantin A., Yan N.D., Cristescu M.E. (2011). An Integrated Multi-Disciplinary Approach for Studying Multiple Stressors in Freshwater Ecosystems: Daphnia as a Model Organism. Integr. Comp. Biol..

[19] Tsui M.T.K., Wang W.-X. (2004). Uptake and Elimination Routes of Inorganic Mercury and Methylmercury in *Daphnia magna*. Environ. Sci. Technol..

[20] Bownik A. (2017). Daphnia swimming behaviour as a biomarker in toxicity assessment: A review. Sci. Total Environ..

[21] Melvin S.D., Wilson S.P. (2013). The utility of behavioral studies for aquatic toxicology testing: A meta-analysis. Chemosphere.

[22] Reif D.M., Truong L., Mandrell D., Marvel S., Zhang G., Tanguay R.L. (2016). High-throughput characterization of chemical-associated embryonic behavioral changes predicts teratogenic outcomes. Arch. Toxicol..

[23] Hansen L.R., Roslev P. (2016). Behavioral responses of juvenile *Daphnia magna* after exposure to glyphosate and glyphosate-copper complexes. Aquat. Toxicol..

[24] Noss C., Dabrunz A., Rosenfeldt R.R., Lorke A., Schulz R. (2013). Three-Dimensional Analysis of the Swimming Behavior of *Daphnia magna* Exposed to Nanosized *Titanium Dioxide*. PLoS ONE.

[25] Untersteiner H., Kahapka J., Kaiser H. (2003). Behavioural response of the cladoceran *Daphnia magna* Straus to sublethal Copper stress—Validation by image analysis. Aquat. Toxicol..

[26] Christensen B.T., Lauridsen T.L., Ravn H.W., Bayley M. (2005). A comparison of feeding efficiency and swimming ability of *Daphnia magna* exposed to cypermethrin. Aquat. Toxicol..

[27] Tkaczyk A., Bownik A., Dudka J., Kowal K., Ślaska B. (2021). *Daphnia magna* model in the toxicity assessment of pharmaceuticals: A review. Sci. Total Environ..

[28] International Organization for Standardization. https://www.iso.org/standard/40518.html.

[29] National Public Service Platform for Standards Information. https://openstd.samr.gov.cn/bzgk/gb/newGbInfo?hcno=CF2E75792CF341FCD45F2613140293D1.

[30] Tsui M.T.K., Wang W.-X. (2006). Acute Toxicity of Mercury to *Daphnia magna* under Different Conditions. Environ. Sci. Technol..

[31] Kim H., Yim B., Bae C., Lee Y.-M. (2017). Acute toxicity and antioxidant responses in the water flea *Daphnia magna* to xenobiotics (cadmium, lead, mercury, bisphenol A, and 4-nonylphenol). Toxicol. Environ. Health Sci..

[32] Monteiro D.A., Rantin F.T., Kalinin A.L. (2010). Inorganic mercury exposure: Toxicological effects, oxidative stress biomarkers and bioaccumulation in the tropical freshwater fish matrinxã, *Brycon amazonicus* (Spix and Agassiz, 1829). Ecotoxicology.

[33] Chen Z., Du M., Yang X.-D., Chen W., Li Y.-S., Qian C., Yu H.-Q. (2023). Deep-Learning-Based Automated Tracking and Counting of Living Plankton in Natural Aquatic Environments. Environ. Sci. Technol..

[34] Ge Z., Liu S., Wang F., Li Z., Sun J. (2021). YOLOX: Exceeding YOLO Series in 2021. arXiv.

[35] Zhang Y., Sun P., Jiang Y., Yu D., Weng F., Yuan Z., Luo P., Liu W., Wang X. ByteTrack: Multi-object Tracking by Associating Every Detection Box. Proceedings of the European Conference on Computer Vision 2022.

[36] Yuan S., Liang C., Li W., Letcher R.J., Liu C. (2021). A comprehensive system for detection of behavioral change of *D. magna* exposed to various chemicals. J. Hazard. Mater..

[37] Richetti S.K., Rosemberg D.B., Ventura-Lima J., Monserrat J.M., Bogo M.R., Bonan C.D. (2011). Acetylcholinesterase activity and antioxidant capacity of zebrafish brain is altered by heavy metal exposure. NeuroToxicology.

[38] Ren Z., Zhang X., Wang X., Qi P., Zhang B., Zeng Y., Fu R., Miao M. (2015). AChE inhibition: One dominant factor for swimming behavior changes of *Daphnia magna* under DDVP exposure. Chemosphere.

[39] Pereira P., Puga S., Cardoso V., Pinto-Ribeiro F., Raimundo J., Barata M., Pousão-Ferreira P., Pacheco M., Almeida A. (2016). Inorganic mercury accumulation in brain following waterborne exposure elicits a deficit on the number of brain cells and impairs swimming behavior in fish (white seabream—*Diplodus sargus*). Aquat. Toxicol..

[40] Teixeira F.B., Fernandes R.M., Farias-Junior P.M.A., Costa N.M.M., Fernandes L.M.P., Santana L.N.S., Silva-Junior A.F., Silva M.C.F., Maia C.S.F., Lima R.R. (2014). Evaluation of the Effects of Chronic Intoxication with Inorganic Mercury on Memory and Motor Control in Rats. Int. J. Environ. Res. Public Health.

[41] Ferrão-Filho A.S., Soares M.C.S., Lima R.S., Magalhães V.F. (2014). Effects of *Cylindrospermopsis raciborskii* (cyanobacteria) on the swimming behavior of Daphnia (*cladocera*). Environ. Toxicol. Chem..

[42] Pawlik-Skowrońska B., Bownik A. (2021). Cyanobacterial anabaenopeptin-B, microcystins and their mixture cause toxic effects on the behavior of the freshwater crustacean *Daphnia magna* (*Cladocera*). Toxicon.

[43] Xuereb B., Lefèvre E., Garric J., Geffard O. (2009). Acetylcholinesterase activity in Gammarus fossarum (*Crustacea Amphipoda*): Linking AChE inhibition and behavioural alteration. Aquat. Toxicol..

